# Analysis of full length transcriptome and resistance characteristics of *Atraphaxis bracteata* under drought

**DOI:** 10.1038/s41598-024-80831-2

**Published:** 2025-01-04

**Authors:** Cai He, Wei Liu, Min Jin, Yi Li, Yuan Wu, Lishan Shan, Peifang Chong, Meiying Wei, Fang Hu, Yuan Liu, Chaoqun Li, Qinde Zhang

**Affiliations:** 1 College of Forestry, Gansu Agricultural University, Lanzhou, 730000 China; 2 Wuwei Academy of Forestry, Wuwei, 733000 China

**Keywords:** *Atraphaxis bracteata*, Full-length transcripts, Differentially expressed transcript, Drought stress, Circadian rhythm-plant, Genetics, Molecular biology, Physiology, Plant sciences

## Abstract

Drought is one of the main environmental factors affecting plant survival and growth. *Atraphaxis bracteata* is a common desert plant mainly utilized in afforestation and desertification control. This study analyzed the morphological, physiological and molecular regulatory characteristics of different organs of *A. bracteata* under drought stress. The results showed that with the intensification of drought stress, the height, root length and leaf area of *A. bracteata* seedlings decreased, while the content of osmotic substances and antioxidant enzyme activity increased. Furthermore, a total of 63 907 non-redundant transcript sequences, of which 55 574 transcripts were annotated, 248 178 FLNC sequences, 107 112 high-quality consistent sequences and 291 314 CCSs were obtained from Iso-Seq. Meanwhile, a total of 2 039 AS events, 22 919 SSR, 40 404 CDS and 5 902 lncRNA were also obtained. The RNA-Seq analysis results revealed that a total of 2 821, 3 907 and 5 532 DETs were identified from roots, stems and leaves, respectively, and which had significantly enrichment in “circadian rhythm-plant” and “starch and sucrose metabolism” pathway. These results would be great significance for further research on the stress resistance of *A. bracteata* and these DETs function.

## Introduction

Drought is one of the main climatic factors that seriously affects most of plants survival and growth^1,2^, which is mainly achieved by influencing photosynthesis, hormone balance and other many key physiological and biochemical processes^3^. When plants were under drought stress, they usually resisted external stress by regulating changed in photosynthesis, relative water content, protective enzyme systems, osmotic substances, etc. However, with the development of molecular biology, it had been found that plants adapt to drought response through a series of morphological, structural and biochemical changed, which were closely related to the relevant gene expression in organisms^4–7^. Desert plants have developed strong resistance on the drought, cold, high temperature, sand burial, salt alkali and other characteristics during their long-term continuous adaptation to extreme climate conditions. They have inevitably formed their unique stress resistance molecular mechanisms and are excellent resources for screening stress resistance genes.

*Atraphaxis bracteata* (A. Los.) is a desert xerophytic broad-leaved shrub of Polygonaceae and *Atraphaxis*, belongs to the main desert afforestation shrub in desert areas, which is not only suitable for growth in drought soil, but also has a certain tolerance to water and humidity^8,9^. In addition, it could grow on the mobile sand dunes in both desert and semi desert areas, with high survival rate, large annual growth, developed root systems and a preference for sand burial, so it was also an excellent fuelwood forest resource in desert areas^10^. In recent years, scholars have also conducted some researches on *A. bracteata* and found that environmental factors had a significant impact on the sap flow of *A. bracteata*, of which solar radiation was the most important environmental factor. During the growth season, the sap flow rate of the tree trunk was relatively high and the daytime sap flow was significantly affected by meteorological conditions^11–13^. Although the transpiration of *A. bracteata* was lower than that of *Elaeagnus angustifolia* and *Corethrodendron scoparium*, it was higher than the typical afforestation tree species *Caragana korshinskii* and *Haloxylon ammodendron* in desert areas^14^. *A. bracteata* had strong adaptability to high temperature, drought, strong light and sandy environments, just like its antioxidant capacity remained at a high level throughout the entire drought stress period based on its strong antioxidant defense system^15–17^, and its leaf structure also exhibited typical characteristics of desert plants and strong drought resistance^18^. In addition, the wild *A. bracteata* community had few biotypes, simple composition and obvious characteristics of desert grassland vegetation, and which also had strong natural regeneration ability and superiority in different habitats. Therefore, the use of *A. bracteata* for desert vegetation restoration and reconstruction would have great significance^19,20^.

With the tremendous growth of science and technology, the transcripts research methods have become diverse and diverse for solving the two major aspects of gene structure and gene function in transcripts^21^. RNA sequencing (RNA-Seq) technology had the advantages of short sequencing time, low cost, high accuracy and high throughput based on Illumina/Solex sequencing compared to traditional sequencing methods^22^. It could also obtain low abundance expressed genes and be used for species research with unknown genome sequences. Therefore, it was widely used in many fields^23,24^. In terms of plant stress resistance, the RNA-seq technology was used to comprehensively and dynamically detect the characteristics of gene expression changes in plants body under stress, excavate some related functional genes, analyze the regulatory mechanism of stress response, and lay on molecular genetic basis for studying plant stress resistance mechanisms and cultivating stress resistant varieties^25–29^.

It can be said that transcriptome has become one of the essential tools for scholars to study life processes. However, because of the intrinsic limitations of RNA-Seq technology, it is challenging for us to comprehend the intricate meaning of life activity because it is unable to reliably obtain or assemble complete transcripts of organisms or recognize transcripts expressed by isomers, homologous genes, superfamily genes and alleles. Full-length cDNA could be obtained by reverse transcription without the need for interrupting RNA fragments in Isoform-Sequencing (Iso-Seq). Measurable information was obtained from the platform’s super long read (median 10 kb), which provided information about a single full transcript sequence and subsequent analysis did not need assembly^30,31^. Currently, Iso-Seq was being applied extensively in numerous fields^32–37^, including studies on drought-resistant plants^33,38–41^.

Using Iso-Seq in conjunction with RNA-Seq, we examined the molecular regulatory mechanism of the desert plant *A. bracteata* in response to drought stress. A total of 63 907 non-redundant full length non-chimeric (FLNC) sequences and 291 314 circular consensus sequences (CCSs) with an average read length of 2 062 were acquired. A total of 2 821, 3 907 and 5 532 differential expressed transcripts (DETs) were identified from CKR vs. HR24h, CKS vs. HS24h and CKL vs. HL24h, respectively. As a result, which would offer fresh perspectives on the molecular biological aspects of *A. bracteata*'s drought resilience as well as a theoretical framework for future research and application.

## Materials and methods

### Plant materials

The experimental materials of *A. bracteata* were obtained from Gansu Desert Control Research Institute, Lanzhou, P.R. China in April 2022 (Delu Li Researcher officially identified the plant materials, lidlu2008@163.com), and which were cultivated at the comprehensive experimental station of Wuwei Academy of Forestry, Wuwei, P.R. China in May 2022. A specimen was deposited at Wuwei Academy of Forestry under the voucher number WWSLKY-HMS-202204–01. The experimental materials used for *A. bracteata* were licensed by the Gansu Desert Control Research Institute and Wuwei Academy of Forestry. The *A. bracteata* seeds with healthy and plump were selected and soaked in clean water for 4 h before planting, and then planted them in the nutrient bowl (10 × 12 cm) which containing some substrate and cultivated in the solar greenhouse (the average temperature was 24 ~ 26 °C, the average humidity was 55 ~ 60%). After the seedlings grew to 40 days, started drought treatment. The sorbic alcohol with osmotic pressure of -0.2 MPa, -0.4 MPa, -0.6 MPa were used to simulate drought treatment, and clear water as the control, and which named T1, T2, T3 and CK, respectively. During treatment, selected the nutrient bowl with basically the same seedling growth and placed them in a tray containing the equal treatment solution.

Once the nutrient bowl had reached saturation with the treatment solution, commenced timing. After 24 h, collected a mixed sample consisting of roots, stems and leaves from the CK, T1, T2 and T3 groups for Iso-Seq analysis. Concurrently, separately collected samples of roots, stems and leaves from the T3 and CK groups for RNA-Seq analysis. Thereafter, maintained the treatment by replenishing the water every three days using the identical treatment protocol. After 15 days, collected the roots, stems and leaves from each sample and stored them at -80°C for the determination of their physiological and biochemical indicators. Additionally, the roots, stems and leaves of T3 and CK used for RNA-Seq analysis were named HR24h, HS24h, HL24h, CKR, CKS and CKL, respectively.

### Measurement of morphological and physiological indicators

A tape and a vernier caliper were used to measure the height, root length and leaf area of each treated plant. The content of malondialdehyde (MDA), proline (Pro), soluble protein (SP), as well as the activities of peroxidase (POD), catalase (CAT) and superoxide dismutase (SOD) in the sample were determined according to the methods in “principles and techniques of plant physiology and biochemistry experiments”.

### RNA preparation

The total RNA was extracted from the experimental materials according to the specifications of manufacturer of the RNA isolation Kit (Tiangen Biotech, Beijing, China) and each sample had 3 biological replicates. To guarantee the quality of transcriptome sequencing samples, 1% agarose gel electrophoresis and LabChip GX were used to assess the quality and integrity of their total RNA.

### Construction and sequencing of PacBio Iso-Seq libraries

Biomarker Technologies Co. Ltd. (Beijing, China) was commissioned for this study to sequence *A. bracteata’s* Iso-Seq and RNA-Seq. The qualified RNA sample were used to construct the Iso-Seq library constructions, the main process was as follows: (1) The NEBNext Single Cell/Low Input cDNA Synthesis & Amplification Module was utilized to synthesize full-length cDNA of mRNA; (2) PCR amplification of full-length cDNA and performing damage repair/end repair on it; (3) Connection of the single molecule real-time sequencing (SMRT) dumbbell. Following cDNA library quality testing, the PacBioSeque II was employed for Iso-Seq according to the intended offline data.

The RNA-Seq library was constructed using the following steps: Eukaryotic mRNA was enriched with Oligo (dT) magnetic beads. The fragmentation buffer was applied to randomly break mRNA. Using the mRNA as a template and random hexamers, the first cDNA strand was produced by random hexamers. The dNTPs, buffer, RNase H and DNA polymerase I were added to create the second strand of cDNA. The AMPure XP beads were utilized to purify the cDNA, which was subsequently corrected and A-tail added before being coupled to sequencing connections. At last, the cDNA library was generated through PCR enrichment.

### Transcriptome raw data analysis

The raw subreads was analyzed according to the Iso-Seq3 pipeline. When conducting full-length transcript analysis, all original sequences were first transformed into CCSs based on the adapters in the sequence, and which were also polished to obtain the quality information of the CCSs. After that, the CCSs were divided to FLNC and non-full-length (NFL) based on the presence or absence of 3’primers, 5'primers and Poly-A tails. Finally, clustering similar sequences from FLNC sequences into a cluster using the Iso-seq3 and extracting high-quality (HQ) isoforms and removing redundancy using cd-hit (identity > 99%). The clean data were extracted from the raw data. Meanwhile, their GC-content, sequence duplication level, Q20 and Q30 were detected, only HQ clean data were selected for subsequent analysis.

### Transcripts function analysis

The obtained non-redundant transcript sequences were compared with non-redundant protein databasse (NR)^42^, swissprot protein database (SWISSPROT) (Consortium)^43^, gene ontology (GO)^44^, clusters of Orthologous Groups (COG)^45^, eukaryotic ortholog groups (KOG)^46^, Pfam (Protein family)^47^ and kyoto encyclopedia of genes and genomes (KEGG) (Kanehisa et al.)^48^ databases by DIAMOND software. InterProScan utilized the InterPro integrated database to analyze the GO methodology results of non-redundant transcript sequences. After calculating amino acid (aa) sequences of non-redundant transcripts, the HMMER software was used to analyze the transcripts annotation information. In addition, GO, COG and KEGG enrichment were taken to further analyze the DETs function.

### Structure analysis

#### CDS detection

TransDecoder was used to detect the candidate coding regions transcript sequences and their corresponding aa sequences. The methods applied were as follows: (1) It was necessary to find an open reading frame (ORF) that satisfies the minimum length in the transcript sequence. (2) When the logarithmic likelihood score of the first reading frame was the greatest compared to the other five reading frames. (3) If the candidate ORF was completely included within the framework of other candidate ORFs, the longest ORF was reported. Otherwise, a single transcript would receive reports of multiple ORFs. (4) As an optional option, the predicted peptides have alignment scores higher than the score threshold in the Pfam domain library.

#### SSR and AS detection

The simple sequence repeats (SSRs) were identified by MISA software, which could identify seven forms of SSRs by evaluating transcript sequences, including mononucleotide, dinucleotide, trinucleotide, tetranucleotide, pentanucleotide, hexanucleotide and compound SSR. In this study, we selected over 500 bp transcripts for further SSR analysis.

Prediction of AS candidate events based on FLNC sequences of Iso-Seq. The BLAST software was used to compare all sequences in pairs, if comparison results met the undermentioned criteria, it was selected as a candidate AS event. (1) Two sequences were over 1000 bp long and included two high-scoring segment pairs (HSPs). (2) The AS gap exceeded 100 bp and located 100 bp from the 3’/5' end at a minimum. (3) Permit 5 bp overlap.

#### Quantification of transcript expression levels and differential expression analysis

In present research, the Iso-Seq data were taken as the reference transcriptome. The expression levels of the transcripts were directly tallied and quantified by using the Kallisto software to compare the readings of the fq file to the third-generation transcripts. Fragments per kilobase of transcript per million fragments mapped (FPKM) was used to estimate the expression level of transcript. The differential expression of two comparison groups was investigated using DESeq2^49^. The fold change (FC) ≥ 2 and false discovery rate (FDR) < 0.01 were taken to select the DETs.

#### Quantitative real-time polymerase chain reaction (qRT-PCR)

qRT-PCR was performed using a quantitative reagent 2X SG Fast qPCR Master Mix (B639271, BBI, Roche) in a LightCycler480 II fluorescence quantitative PCR instrument (Roche, Rotkreuz, Switzerland). Reaction system: 2X SG Fast qPCR Master Mix 5 µL, 10 µM Primers F and R each 0.2 µL, ddH20 3.6 µL, Template (cDNA) 1 µL. The qRT-PCR values were calculated using the comparative cycle threshold method 2^−(∆∆ Ct)^ method, with 3 technical replicates for each sample. The specific gene and internal reference gene (PB-MIX_transcript_58967) primers were shown in Table S1.

## Results

### The effect of drought stress on the phenotype of *A. bracteata*

**Fig. 1:**
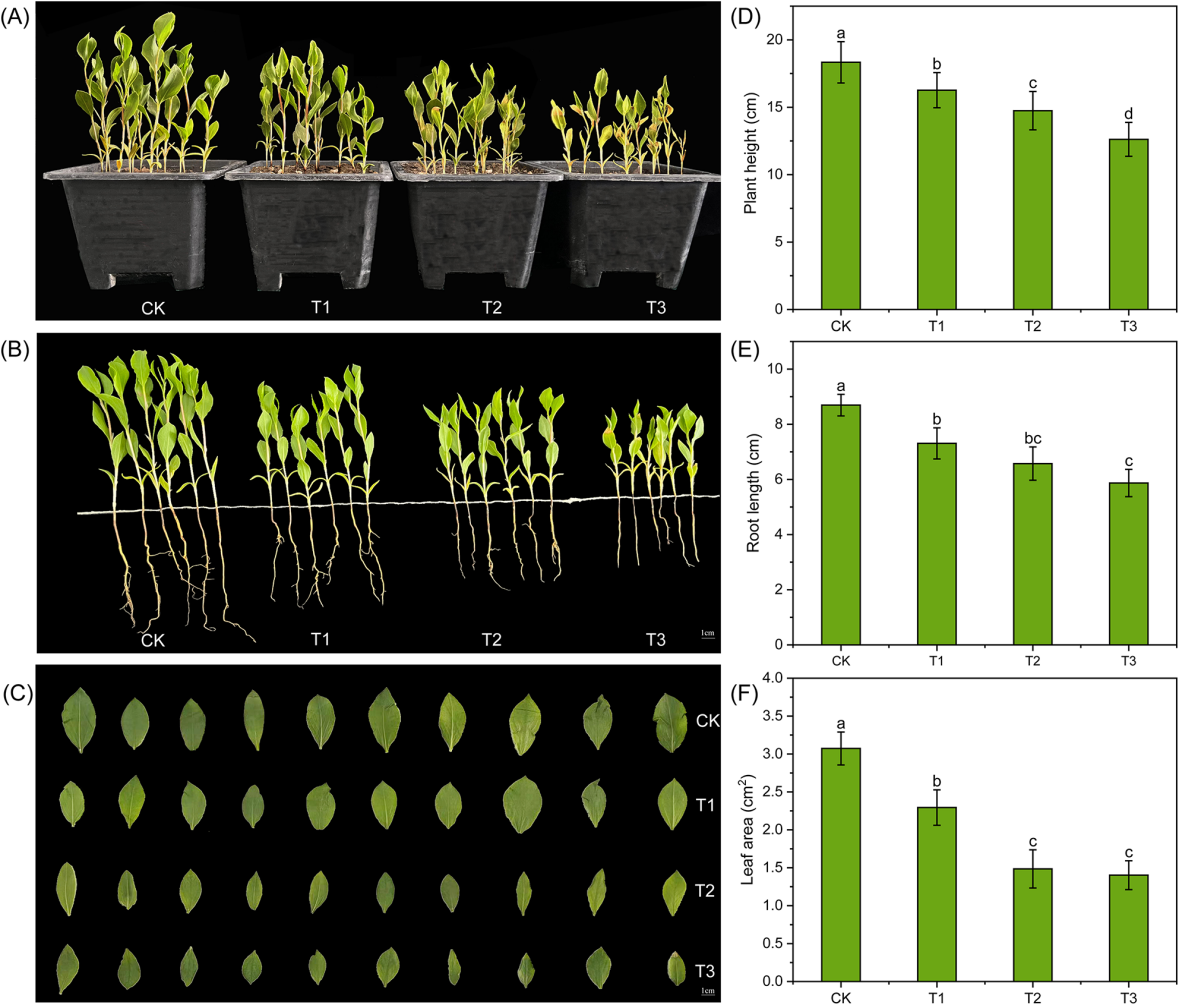
The phenotype of *A. bracteata* under drought stress. (**A**) , (**B**) and (**D**) The effect of drought stress on the growth of *A. bracteata*. (**B**) and (**E**) The effect of drought stress on the roots of *A. bracteata*. (**C**) and (**F**) The effect of drought stress on the leaves of *A. bracteata*.

After 15 days of simulated drought treatment with sorbitol, significant phenotypic changes occurred between the treatments and the control, mainly manifested as slower plant growth and smaller leaf area. Analysis of plant height showed that after drought stress, the height of each treatment was lower than CK, and there were significant differences between them, indicating that different degrees of drought stress treatment with sorbitol had a certain inhibitory effect on the growth of *A. bracteata* plants, and the degree of inhibition increased with the increased of stress level (Fig. 1A, B, D). By analyzing the leaf area of each treatment, it was found that as the degree of drought stress increased, the leaf area gradually decreased, and there were significant differences between each treatment and CK. The treatment T1 had significant differences with T2 and T3, while there was no significant difference between T2 and T3 (Fig. 1C, F). In addition to changes in the phenotype of above ground organs, significant changes were also observed in their underground roots. Root length was significantly inhibited between treatments compared to CK, but except for T1 and T3 had significantly differences, there were no significant differences among other drought treatments (Fig. 1B, E).

### The effect of drought on physiological indicators of *A. bracteata*

**Fig. 2:**
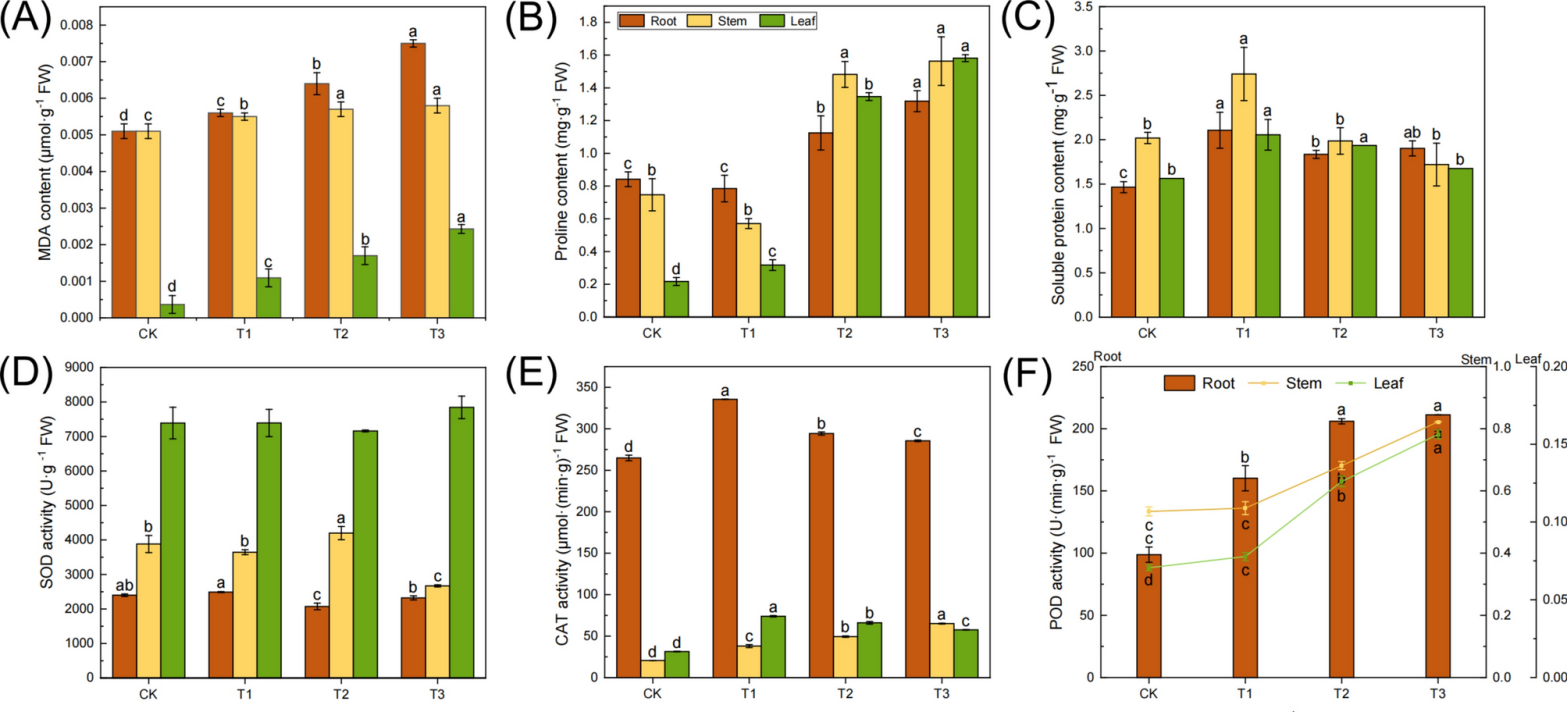
The physiological indicators of *A. bracteata* under drought stress. (**A**) The MDA content. (**B**) The Pro content. (**C**) The SP content. (**D**) The activity of SOD. (**E**) The activity of CAT. (**F**) The activity of POD.

The Fig. 2 illustrated the variation characteristics of the main physiological indicators in different organs of *A. bracteata* under drought treatment. The MDA content ranged from 0.0003 to 0.0075 µmol·g^-1^, with the highest levels in roots, followed by stems and then leaves, and which increased with the degree of drought stress (Fig. 2A). As the degree of drought stress increased, the content of Pro gradually increased, while the SP showed a trend of first increasing and then decreasing (Fig. 2B, C). The Fig. 2D–F showed that the POD and CAT activities in the roots were significantly higher than those in the stems and leaves, and the SOD activities were the highest in leaves. Meanwhile, there was no significant trend in SOD activity, while CAT first increased and then decreased (except for those with an increasing trend in the stems), and POD gradually increased trend. These results indicated that different organs of *A. bracteata* had their own response mechanisms to drought stress, and they could adapt to drought stress by regulating the content of osmotic substances and the activity of antioxidant enzymes.

### Iso-Seq data analysis

A total of 31.86 Gb data were obtained from *A. bracteata* full-length transcriptome, and 291 314 CCSs with an average reading length of 2 062 and a reading base of 600 805 240. In addition, a total of 248 178 FLNC sequences with the percent of 85.19% were obtained after the Iso-Seq polishing. Clustering of FLNC sequences resulted in 107 138 consensus isoforms with an average length of 1 815 and 107 112 high-quality (HQ) consistent sequences with the percent of 99.98%. In addition, a total of 63 907 non-redundant transcript sequences were acquired for further subsequent analysis (Table 1). Contributed raw sequence data to the national center for biotechnology information (NCBI) sequence read archive (SRA), accession number PRJNA 1 028 835.

**Table 1:** The Iso-Seq data information of *A. bracteata.*

Categories	Datasets
Total bases (Gb)	31.86
cDNA size	1-6 K
CCS number	291 314
Read bases of CCS	600 805 420
Mean read length of CCS	2 062
Mean Number of Passes	37
Number of FLNC	248 178
Full-length non-chimeric percentage (FLNC%)	85.19
Number of consensus isoforms	107 138
Average consensus isoforms read length	1 851
Number of high- quality isoforms	107 112
Number of low-quality isoforms	26
Percent of high-quality isoforms (%)	99.98%
Number of Non-redundant transcript sequence	63 907

### Transcript function annotation

**Fig. 3:**
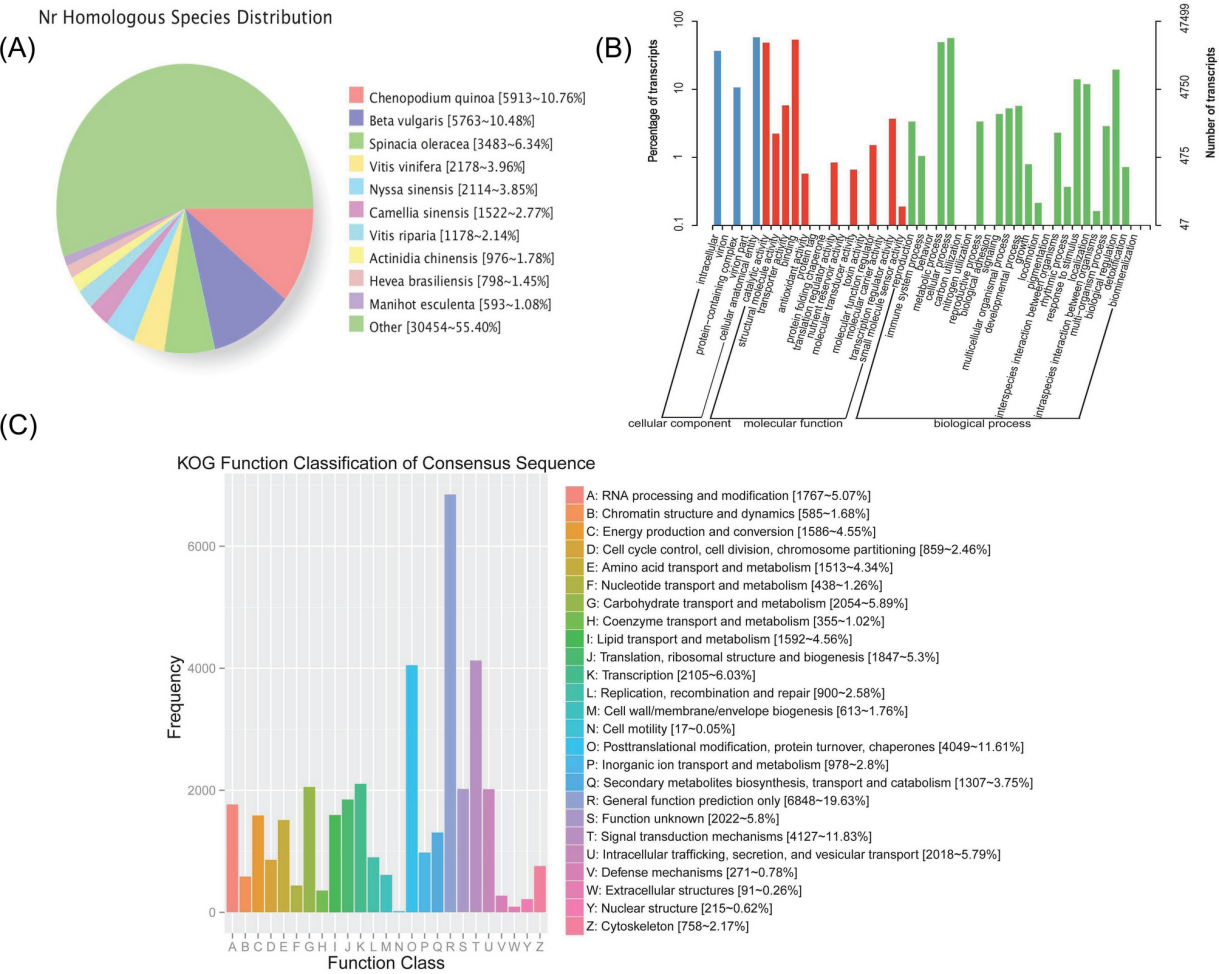
The functional annotation results of the full-length transcripts of *A. bracteata*. (**A**) Statistical diagram of species classification annotated with NR of the full-length transcripts. Different colors represented different species. (**B**) Statistical diagram of GO annotation classification of the full-length transcripts. The horizontal axis represented GO classification, the left side of the vertical axis represented the percentage of transcripts, and the right side represented the number of transcripts. (**C**) Statistical diagram of KOG annotation classification of the full-length transcripts. The horizontal axis represented the classification content of KOG, and the vertical axis represented the number of transcripts.

All 63 907 non-redundant transcript were contrasted to NR, COG, GO, eggNOG, KOG, Pfam, Translation of EMBL (TrEMBL), Swissprot and KEGG databases using DIAMOND software, a total of 55 574 transcripts were annotated, with an annotation rate of 86.96% (Table 2). However, there were still 8 333 transcripts that have not been annotated, which suggested that these unannotated transcripts may be specific transcripts of *A. bracteata* and have high development and utilization value. In addition, we searched for homologous species through the NR database, comparison results displayed that seven most abundant species were *Chenopodium quinoa* (5 913 ~ 10.76%), *Beta vulgaris* (5 763 ~ 10.48%), *Spinacia oleracea* (3 483 ~ 6.34%), *Vitis vinifera* (2 178 ~ 3.96%), *Nyssa sinensis* (2 114 ~ 3.85%), *Camellia sinensis* (1 522 ~ 2.77%) and *Vitis riparia* (1 178 ~ 2.14%), respectively. However, the proportion of transcript sequences that could annotate homology was only 44.6%, and another 55.4% of transcript sequences still have not annotated homologous species. This indicated that excavating the genes related to the characteristics of *A. bracteata* play an important role in further searching for its homologous species (Fig. 3A).

**Table 2:** The number of annotated transcripts in different databases.

Annotated databases	Isoform Number	300 < = length < 1000	length > = 1000
COG	17 581	2 096	15 479
GO	47 499	7 579	39 821
KEGG	40 398	5 886	34 446
KOG	34 880	5 062	29 767
Pfam	42 074	5 726	36 330
Swissprot	41 871	6 378	35 412
TrEMBL	54 880	9 207	45 555
eggNOG	48 679	7 848	40 761
NR	54 972	9 218	45 637
All	55 574	9 417	46 014

We mainly analyzed the functional annotation results of GO and KOG. The GO function annotation results showed that these transcripts were mainly divided into molecular function (MF), cellular component (CC) and biological process (BP), and most of which belonged to BP. There were three major enrich terms related to cellular anatomical entity, intracellular and protein-containing complex in the CC, whereas biological regulation, metabolic processes and cellular processes were notable enriched in the BP, and binding, transporter activity and catalytic activity were mainly enriched in the MF (Fig. 3B). In addition, biological-process (GO: 0,008,150), cellular process (GO: 0,009,987), metabolic process (GO: 0,008,152), cellular metabolic process (GO: 0,044,237), organic substance metabolic process (GO: 0,071,704), biological regulation (GO: 0,065,007) and primary metabolic process (GO: 0,044,238) were significantly enriched in the category of BP, which unigene number were all over 4 000 (Table S2). The KOG enrichment results showed that these transcripts were classified to 25 functional clusters, with “general function prediction only” (6 848, 19.63%), “signal transduction mechanisms” (4 127, 11.83%), and “posttranslational modification, protein turnover, chaperones” (4 049, 11.61%) were for the top three, accounting for a cumulative proportion of 43.07% (Fig. 3C).

### Structure analysis

#### Prediction analysis of CDS and SSR

**Fig. 4:**
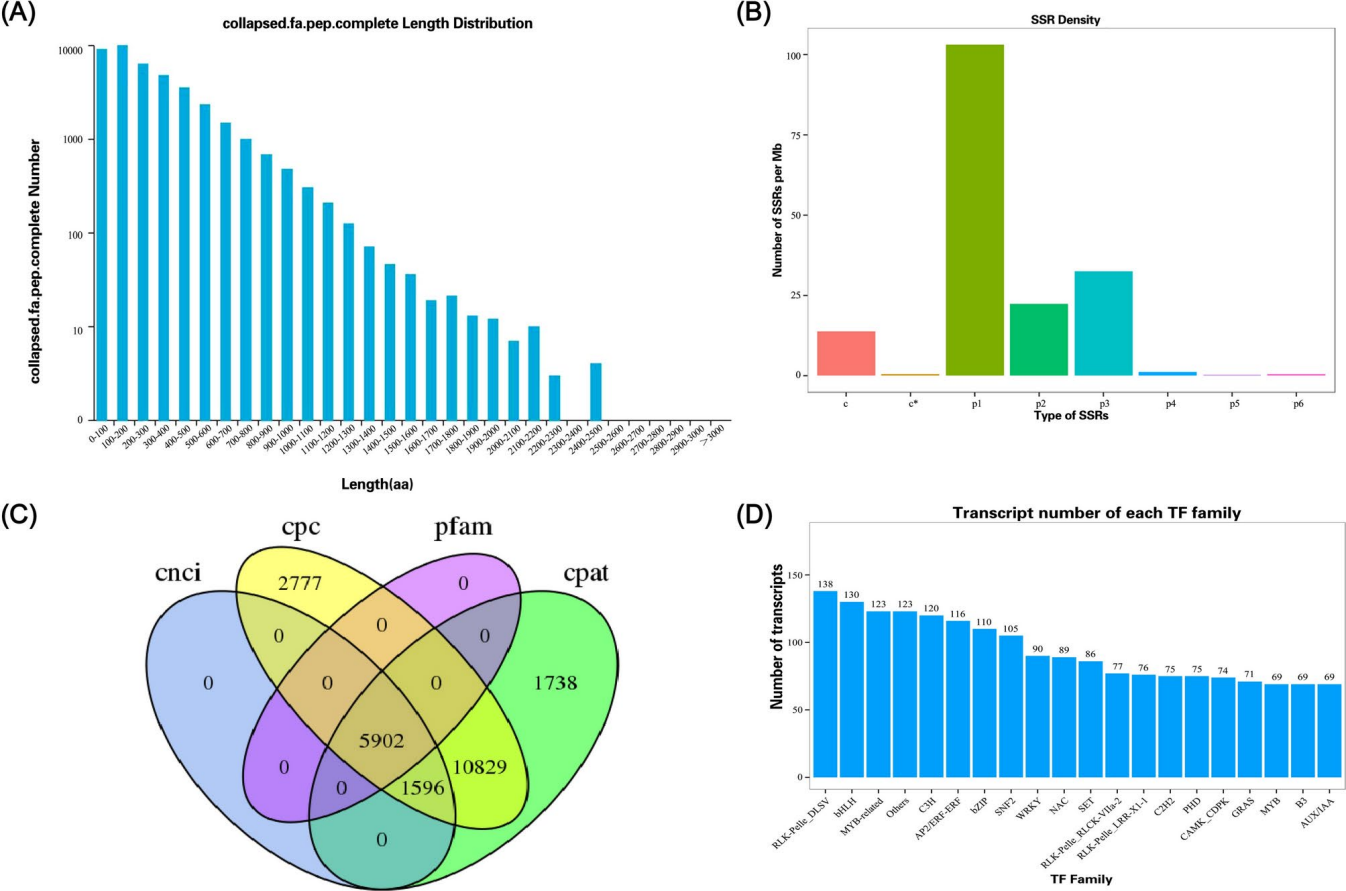
The structure analysis of the full-length transcripts of *A. bracteata*. (**A**) The length distribution diagram of prediction CDS encoded protein. (**B**) Density distribution statistics of different SSR types. (**C**) The venn diagram of lncRNA predicted by CNCI, CPAT, CPC and Pfam. (**D**) The prediction classification chart of transcription factors.

In all, 58 427 ORFs were obtained in this study, including 40 404 complete ORFs which had start and stop codons. In addition, most of the predicting complete ORF region encoded protein sequences were less than 700 aa in length with 37 379 isoforms (92.51%), 2 147 isoforms (5.31%) ranged between 700–1 000 aa, and only 878 (2.17%) isoforms were longer than 1000 aa (Fig. 4A, Table S3).

SSR analysis was performed out applying the MISA software on a subset of transcripts containing upwards of 500 bp, which revealed a total of 20 868 SSRs. The results showed that seven types SSR were identified including mononucleotide repeats (density: 103.12), dinucleotide repeats (density: 22.27), trinucleotide repeats (density: 32.61), tetranucleotide repeats (density: 1.22), pentanucleotide repeats (density: 0.4), hexanucleotide repeats (density:0.56) and compound SSR (density:13.82) (Fig. 4B).

#### AS events, LncRNA and TF identification.

After redundancy elimination, the full-length transcriptome was used to predict AS events. A total of 2 093 AS events were recognized (Table S4)*.* Unfortunately, the kinds of AS events in *A. bracteata* could not be identified because it did not have the reference genome.

To predict long non-coding RNA (lncRNA) in transcripts, we employed the most comprehensive coding potential analytic techniques, primarily the coding potential calculator (CPC), coding-non-coding index (CNCI), pfam protein domain and coding potential assessment tool (CPAT) analyses. The result showed that there were 7 498, 21 104, 5 902 and 20 065 isoforms in the CNCI, CPC, Pfam and CPAT, respectively. Combining the analysis results of four methods, a total of 5 902 lncRNAs were predicted, including 4 788 novel lncRNAs (81.13%) and 1 114 new lncRNAs (18.87%) (Fig. 4C, Table S5).

Transcription factors (TFs) were identified and categorized using the animalTFDB 3.0 and a total of 4 802 TFs were obtained. Meanwhile, the top 10 TF families were RLK-Pelle-DLSV, bHLH, others, MYB-related, C3H, AP2/ERF-ERF, bZIP, SNF2, WRKY and NAC. Among them, “RLK-Pelle-DLSV” family had the most members (138, 2.87%), followed by the “bHLH” family (130, 2.71%), and the third was “others” and “MYB-related” family with 123 members (Fig. 4D, Table S6).

### The transcript expression analysis of *A. bracteata* under drought

#### Comparison of Iso-seq and RNA-seq

In present study, the spliced transcripts alignment to reference (STAR) was used to compare clean reads with transcripts to obtain positional information on the transcripts. The comparative results demonstrated that the percentage of reads mapped to transcripts loci in clean reads were all greater than 80%, and unique mapped reads accounting for the highest proportion, followed by reads mapped to multiple loci, and reads mapped to too many loci accounting for the lowest proportion (Table 3). These results indicated that the RNA-Seq results were reliable and could be used for subsequent analysis.

**Table 3:** The data comparison results between RNA-Seq and Iso-Seq.

Sample ID	Total reads	% of reads mapped to uniquely loci	% of reads mapped to multiple loci	% of reads mapped to too many loci	Mapped statistics
CKR-1	22 303 062	48.71%	34.76%	0.51%	83.98%
CKR-2	21 292 208	50.20%	35.13%	0.55%	85.88%
CKR-3	20 610 836	49.15%	34.72%	0.59%	84.46%
CKS-1	20 824 428	49.53%	35.08%	0.94%	85.55%
CKS-2	20 525 921	46.37%	35.62%	2.76%	84.75%
CKS-3	20 030 625	48.59%	35.12%	0.99%	84.70%
CKL-1	20 344 166	46.85%	35.52%	2.24%	84.61%
CKL-2	20 374 733	48.08%	36.00%	1.36%	85.44%
CKL-3	21 967 515	48.58%	36.07%	1.56%	86.21%
HR24h-1	21 201 728	49.14%	34.95%	0.59%	84.68%
HR24h-2	23 496 024	49.34%	35.43%	0.51%	85.28%
HR24h-3	21 503 534	48.83%	34.49%	0.51%	83.83%
HS24h-1	21 097 545	47.65%	36.39%	0.92%	84.96%
HS24h-2	23 769 730	48.41%	36.18%	0.48%	85.07%
HS24h-3	20 954 926	48.42%	35.72%	0.49%	84.63%
HL24h-1	21 387 246	47.46%	38.13%	0.75%	86.34%
HL24h-2	22 557 998	46.05%	36.72%	1.97%	84.74%
HL24h-3	20 306 887	47.25%	35.70%	0.55%	83.50%

#### Transcript and expression quantification analysis of RNA-Seq

In order to clarify the transcripts expression pattern, the RNA-Seq was used to analyze the expression transcripts of different parts of *A. bracteata* under drought. A total of 384 549 112 clean reads were obtained from 18 samples which included three biological replicates in each group (Table 4). The Q20 of 18 samples was > 97.87%, the Q30 was > 93.9%, and the GC content ranged from 46.32% to 48.34% (Table 2), which showed that the RNA-Seq data was trustworthy and appropriate for additional study. Contributed raw sequence data to the NCBI SRA, accession number PRJNA 1 030 753.

**Table 4:** The evaluation statistical table of RNA-Seq data.

Sample ID	Read number	Base number	GC (%)	Q20 (%)	Q30 (%)
CKR-1	22 303 062	6 674 759 940	46.44	98.09	94.56
CKR-2	21 292 208	6 376 208 880	46.91	97.97	94.12
CKR-3	20 610 836	6 170 104 132	46.32	97.87	93.90
CKS-1	20 824 428	6 233 818 162	46.79	98.27	94.91
CKS-2	20 525 921	6 143 561 570	47.14	98.13	94.58
CKS-3	20 030 625	5 996 556 924	46.86	98.14	94.59
CKL-1	20 344 166	6 088 844 094	48.17	98.01	94.23
CKL-2	20 374 733	6 099 525 456	47.71	98.27	94.91
CKL-3	21 967 515	6 576 086 594	48.34	97.87	93.97
HR24h-1	21 201 728	6 346 531 032	46.59	98.12	94.58
HR24h-2	23 496 024	7 032 866 094	46.48	98.09	94.47
HR24h-3	21 503 534	6 437 821 450	46.57	98.07	94.42
HS24h-1	21 097 545	6 315 409 508	46.92	98.15	94.62
HS24h-2	23 769 730	7 115 330 912	46.77	98.00	94.23
HS24h-3	20 954 926	6 271 772 248	46.95	98.03	94.26
HL24h-1	21 387 246	6 401 988 224	47.51	98.19	94.71
HL24h-2	22 557 998	6 753 289 962	47.74	98.15	94.59
HL24h-3	20 306 887	6 078 726 308	47.47	98.02	94.36

### DETs analysis

**Fig. 5:**
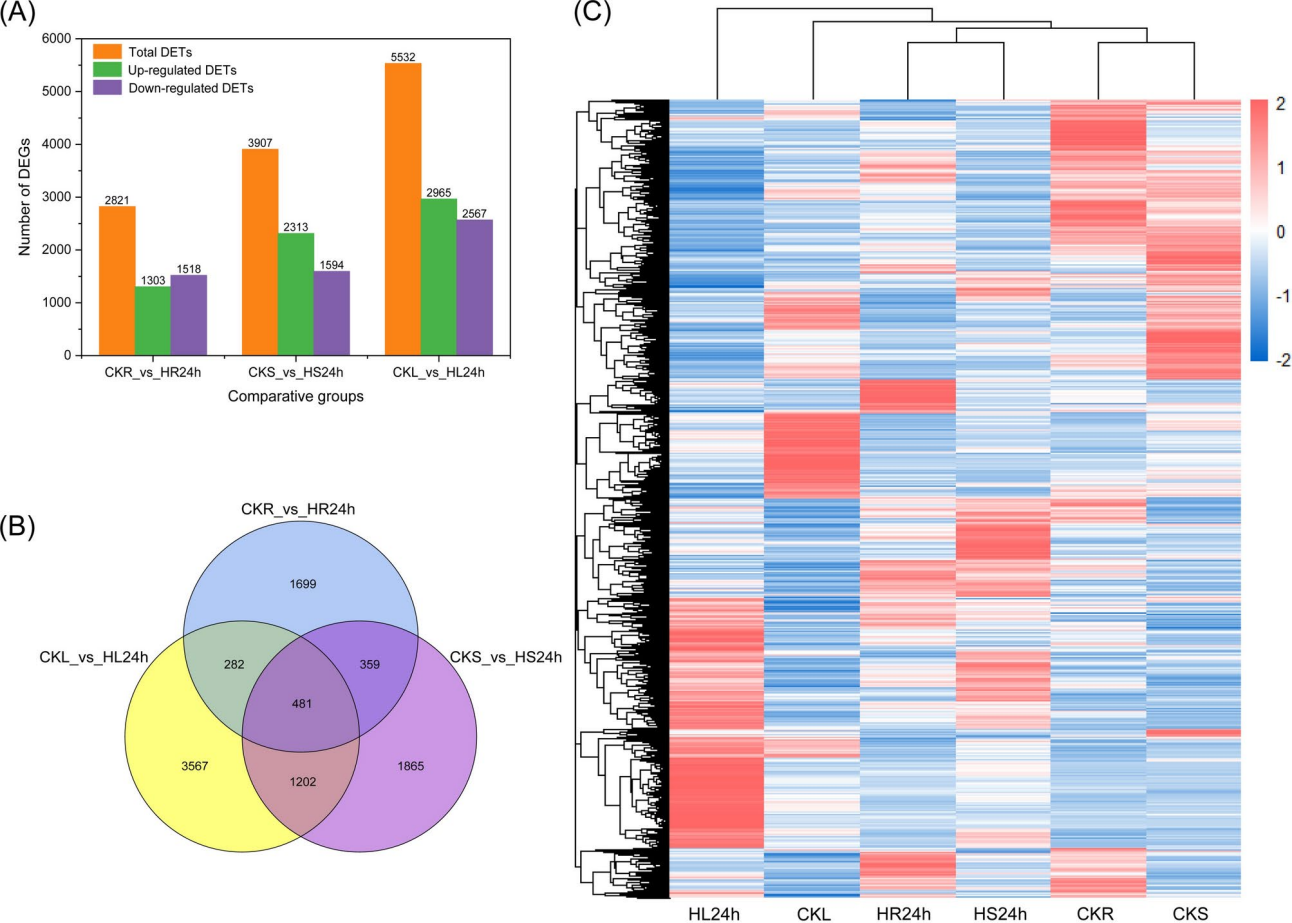
The number analysis of DETs in different organs of *A. bracteata* under CK and drought treatment. (**A**) Statistical histogram of the number of DETs in different organs. (**B**) The venn diagram of DETs in different organs. (**C**) The cluster heat map of DETs in six samples.

In this study, the screening conditions were set as FDR < 0.01 and FC ≥ 2 to obtain DETs, and the statistical results of DETs in each comparison group were shown in Fig. 5A. According to the findings, there were 2 821 DETs in the CKR vs. HR24h, including 1 303 up-regulated and 1 518 down-regulated transcripts. A total of 3 907 DETs were identified from CKS vs. HS24h, including 2 313 up-regulated and 1 594 down-regulated transcript. Meanwhile, 5 532 DETs were identified from CKL vs. HL24h, of which there were 2 965 up-regulated and 2 567 down-regulated transcripts. A total of 481 identical DETs were identified from roots, stems and leaves of *A. bracteata* under drought treatment. In addition, there were also have some unique DETs in different organs, such as a maximum number of unique DETs in leaves with 3 567, followed in stems with 1 865, and a minimum in roots with 1 699 (Fig. 5B). By further analyzing the cluster heat map of these DETs, we found that they were mainly clustered into nine categories and had significant differences in six samples (Fig. 5C). This indicated that the transcripts expression level in different organs of *A. bracteata* had significantly differences, and its leaves were more sensitive to drought stress, followed by the stems and finally the roots.

### The DETs function analysis

To further analysis the functions of DETs in different organs of *A. bracteata* under drought stress, the cluster of COG classification statistics, the GO and KEGG enrichment analysis were used. The “carbohydrate transport and metabolism”, “posttranslational modification, protein turnover, chaperones”, “signal transduction mechanisms”, “general function prediction only” and “amino acid transport and metabolism” were the top five functional clusters of three comparative groups in the COG. However, the frequency of each functional clusters in three comparative groups were significantly differences (Fig. S1). This indicated that different organs of *A. bracteata* may adapt to drought stress by regulating the frequency of DETs in various functional clusters.

**Fig. 6:**
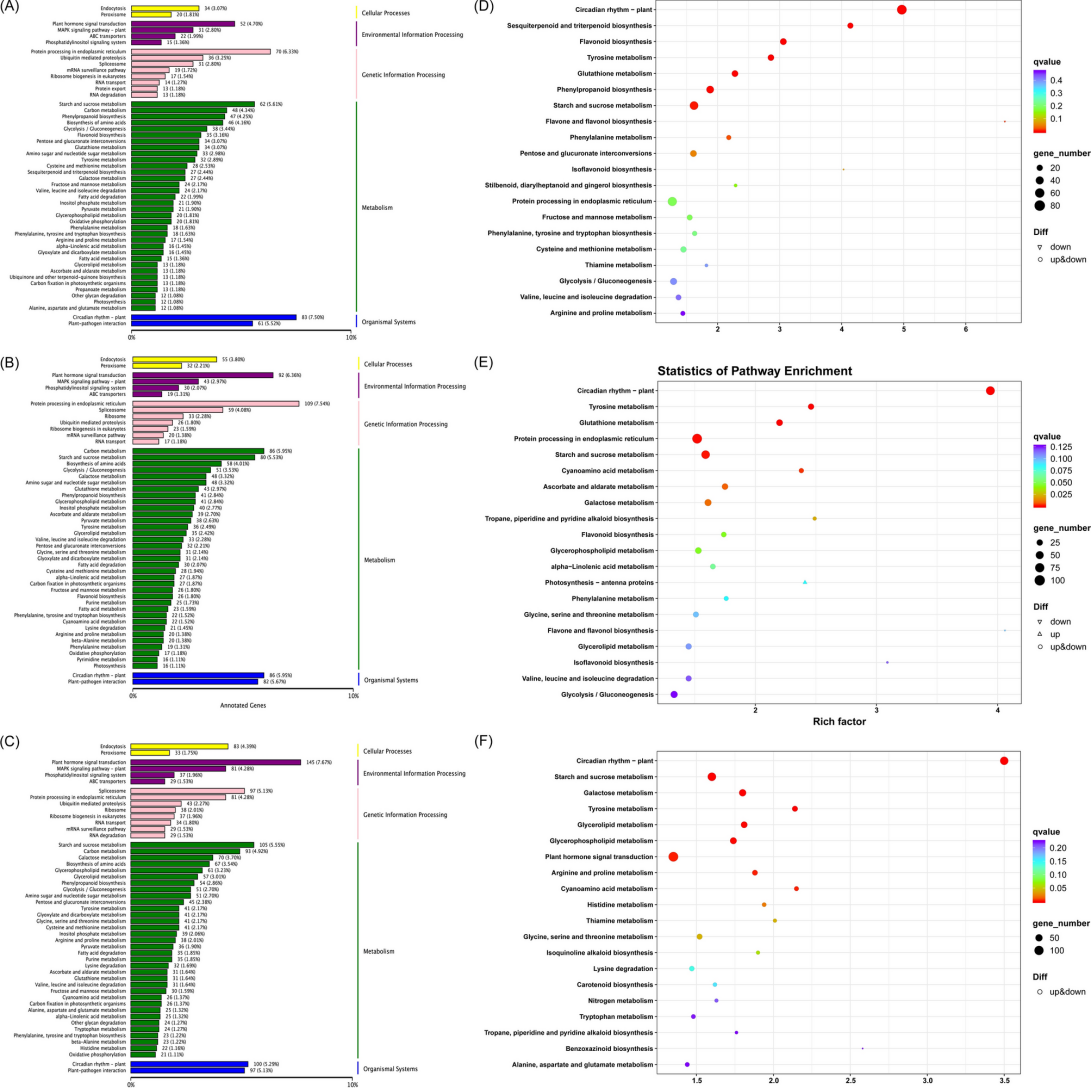
The function analysis of differential expressed transcripts (DETs) in different organs of *A. bracteata* under CK and drought treatment. (**A**)—(**C**): The KEGG annotation classification diagram of DETs. (**A**) CKR vs. HR24h. (**B**) CKS vs. HS24h. (**C**) CKL vs. HL24h. (**D**)—(**F**): The KEGG pathway enrichment diagram of DETs. (**D**) CKR vs. HR24h. (**E**) CKS vs. HS24h. (**F**) CKL vs. HL24h.

For GO enrichment, a total of 2 201 (998 up-regulated, 1 203 down-regulated), 2 986 (1 681 up-regulated, 1 305 down-regulated), and 4 115 (2 041 up-regulated, 2 074 down-regulated) DETs were enriched in CKR vs. HR24h、CKS vs. HS24h and CKL vs. HL24h, respectively. Concurrently, they were significantly enriched to 47, 52, and 51 GO items in CKR vs. HR24h、CKS vs. HS24h and CKL vs. HL24h, respectively. The top five items in the BP were followed by “cellular process”, “metabolic process”, “response to stimulus”, “single-organism process”, and “biological regulation” (Fig. S2). At the same time, KEGG annotation and pathway enrichment were used to analyse the potential biological functions of these DETs. The KEGG annotation results showed that these DETs were mainly divided into 5 categories, namely “cellular processes”, “environmental information processing”, “genetic information processing”, “metabolism” and “organismal systems”, and most of which involved in “metabolism”. Further analysis of the “metabolism” showed that the top three pathways of these DETs participation were followed by “starch and sucrose metabolism” (62, 5.61%), “carbon metabolism” (48, 4.34%) and “phenylpropanoid biosynthesis” (47, 4.25%) in the CKR vs. HR24h, “carbon metabolism” (86, 5.95%), “starch and sucrose metabolism” (80, 5.53%), and “biosynthesis of amino acids” (58, 4.01%) in the CKS vs. HS24h, “starch and sucrose metabolism” (105, 5.55%), “carbon metabolism” (93, 4.92%) and “galactose metabolism” (70, 3.7%) in the CKL vs. HL24h, respectively (Fig. 6 A-C). To further analysed whether there was a significant difference in DETs on a certain pathway, we conducted KEGG pathway enrichment analysis. The enrichment results showed that “circadian rhythm-plant” (ko04712), “starch and sucrose metabolism” (ko00500) and “phenylpropanoid biosynthesis” (ko00940) in the CKR vs. HR24h, “protein processing in endoplasmic reticulum” (ko04141), “circadian rhythm-plant” and “starch and sucrose metabolism” in the CKS vs. HS24h, and “plant hormone signal transduction”, “starch and sucrose metabolism” and “circadian rhythm-plant” in the CKL vs. HL24h were the top three significant enrichment pathway, respectively. Among which, “starch and sucrose metabolism” and “circadian rhythm-plant” were all significantly enriched in three different parts of *A. bracteata* (Fig. 6D-F).

### Analysis of DETs involved in circadian rhythm-plant

There were 83, 86, and 100 DETs in CKR vs. HR24h, CKS vs. HS24h, and CKL vs. HL24h of the circadian rhythm-plant pathway, respectively (Table S7). However, *CRY*, *LHY* and *CDF1* were significantly up-regulated in three comparison groups, while *PHYA* was only up-regulated in the CKR vs. HR24h, PHYB was up-regulated in the CKR vs. HR24h and CKS vs. HS24h, and *PRR9* was up-regulated in the CKS vs. HS24h and CKL vs. HL24h, respectively. Other genes in the circadian rhythm-plant pathway which DETs involved in were down-regulated, such as *PRR7*, *PRR5*, *TOC1*, *GI*, *ELF3*, *PIF3* and *FKF1*. These results indicated that the variation of circadian rhythm-plant pathway caused by DETs in the different organs of *A. bracteata* under drought stress was diverse, and most of them were negatively regulated (Table 5). Further analyzed the functions of these DETs, we found that most of up-regulated DETs which related to *CDF1* and *CHY* belonged to “C2C2-Dof” and “MYB-related” family, respectively, and they had a large number of DETs and a high expression level. Most of down-regulated DETs which related to TFs belonged to “other” and “Pseudo ARR-B” family (Table S6). This indicated that “MYB-related”, “C2C2-Dof”, “other” and “Pseudo ARR-B” of TFs members may play an important role in circadian rhythm-plant pathway of *A. bracteata* under drought stress.

**Table 5:**
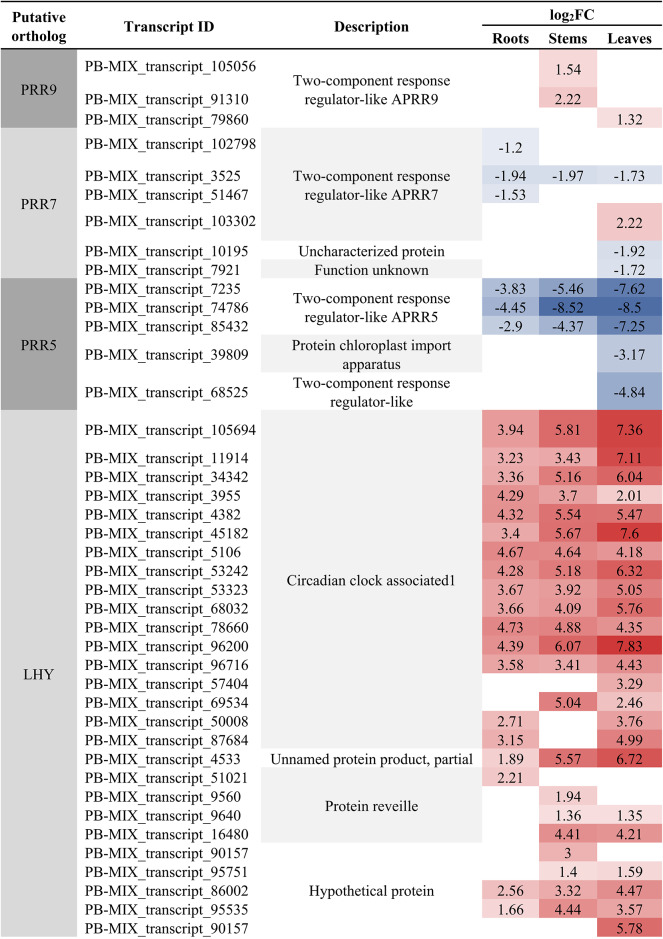

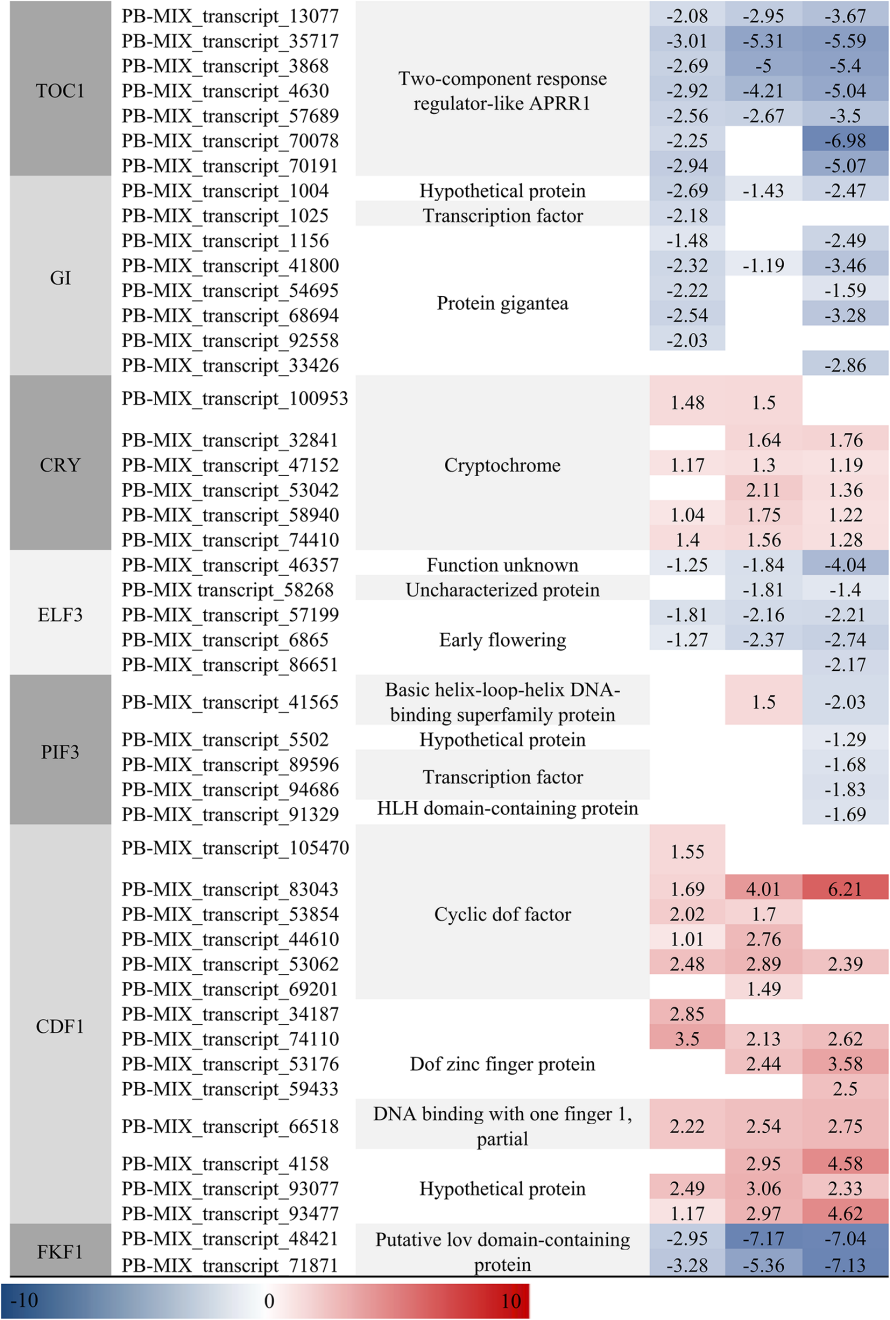
The partial DETs related to circadian rhythm-plant pathway.

### Analysis of DETs involved in arginine and proline metabolism

In the arginine and proline metabolism, a total of 17, 20, and 38 DETs were identified in CKR vs. HR24h, CKS vs. HS24h, and CKL vs. HL24h, respectively. Most of these DETs were up-regulated, and the number of DETs was highest in leaves, followed by stems, and lowest in roots. In addition, some DETs involved in the regulation of proline synthesis genes “*PRODH*, *fadM, putB*, *rocD*, *OAT*, *ALDH18A1*, *P5CS*, *P4HA*” were all up-regulated, and their numbers was also the highest in leaves, and less in roots and stems (Table 6).

**Table 6:**
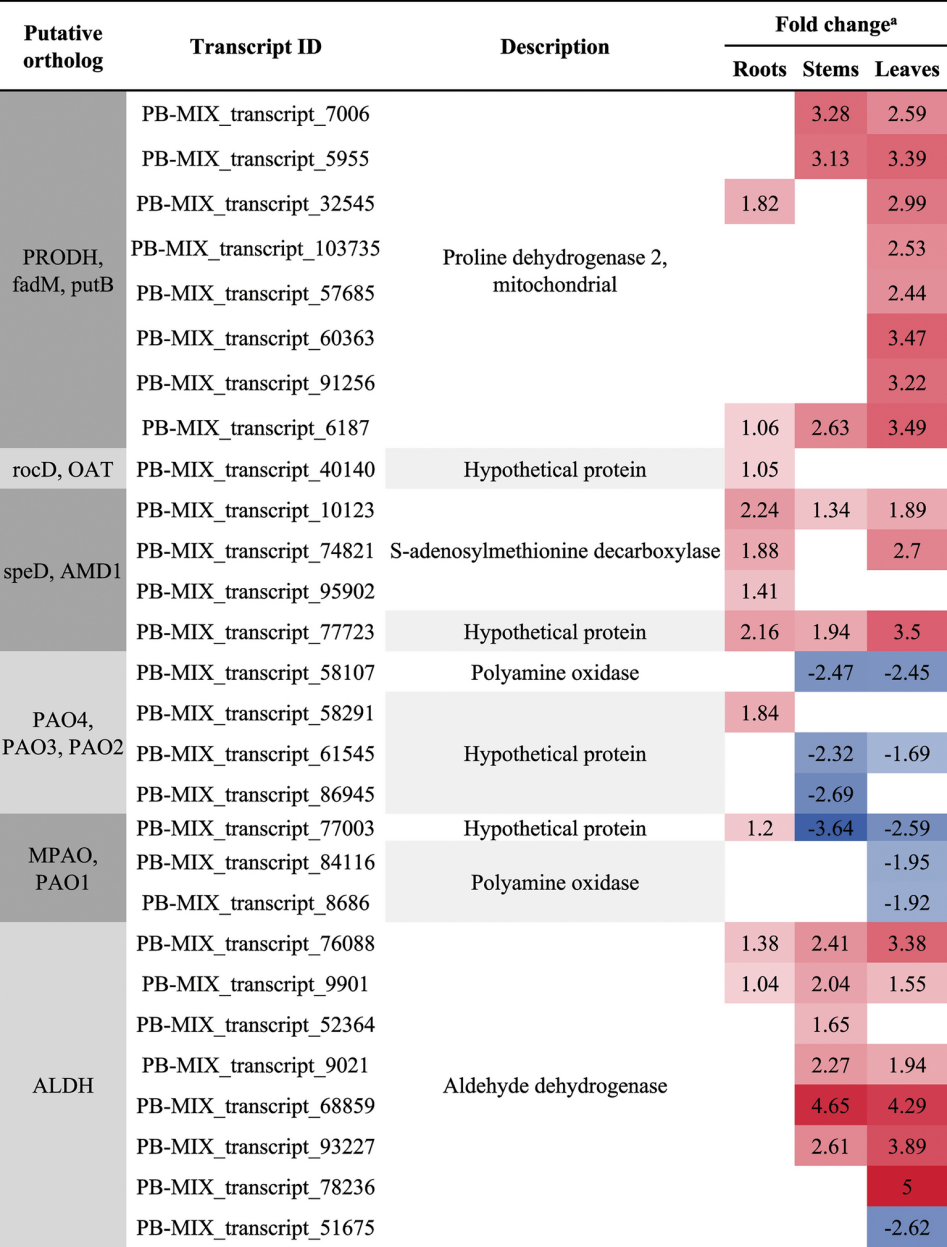

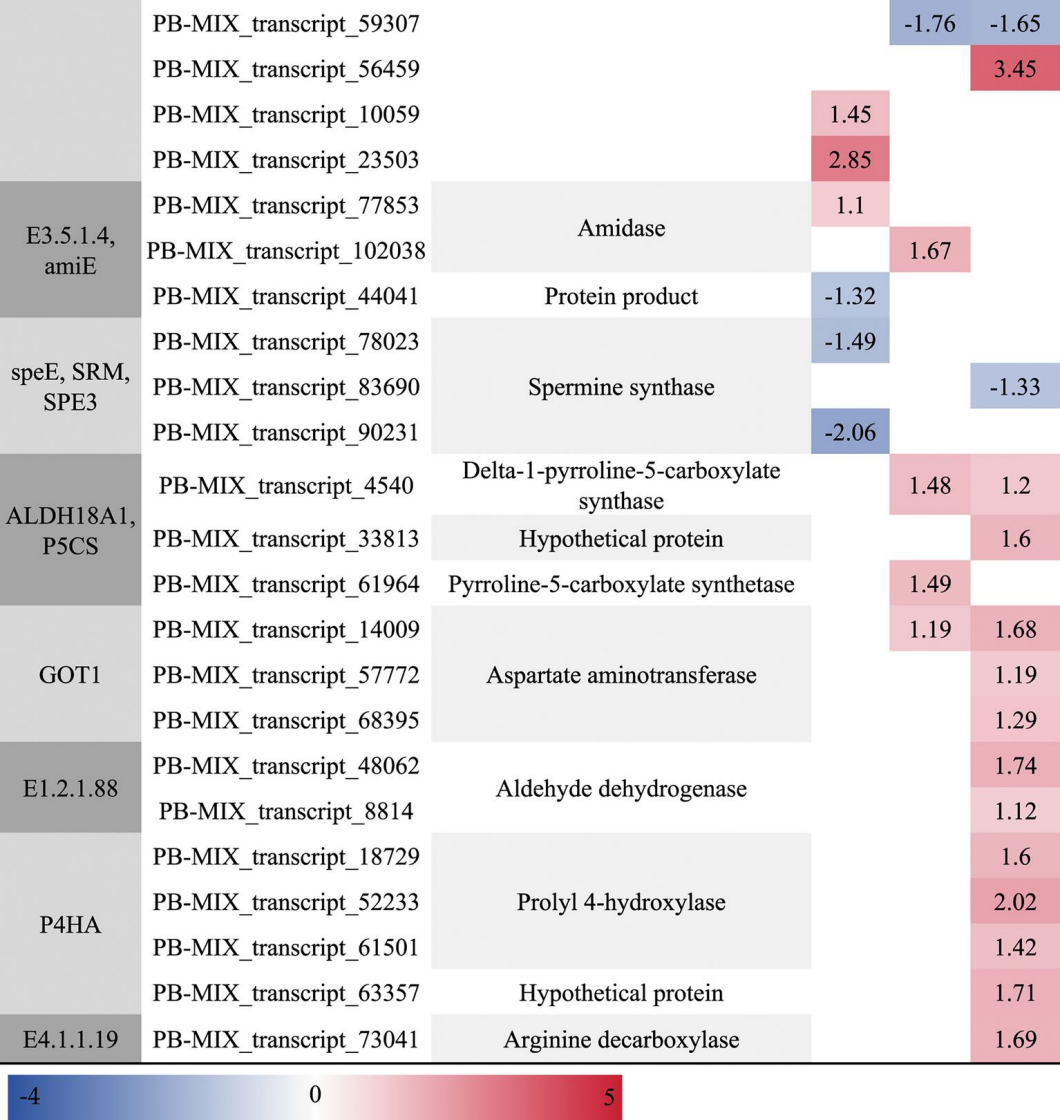
The DETs related to arginine and proline metabolism.

### qRT-PCR validation of transcriptome

**Fig.7:**
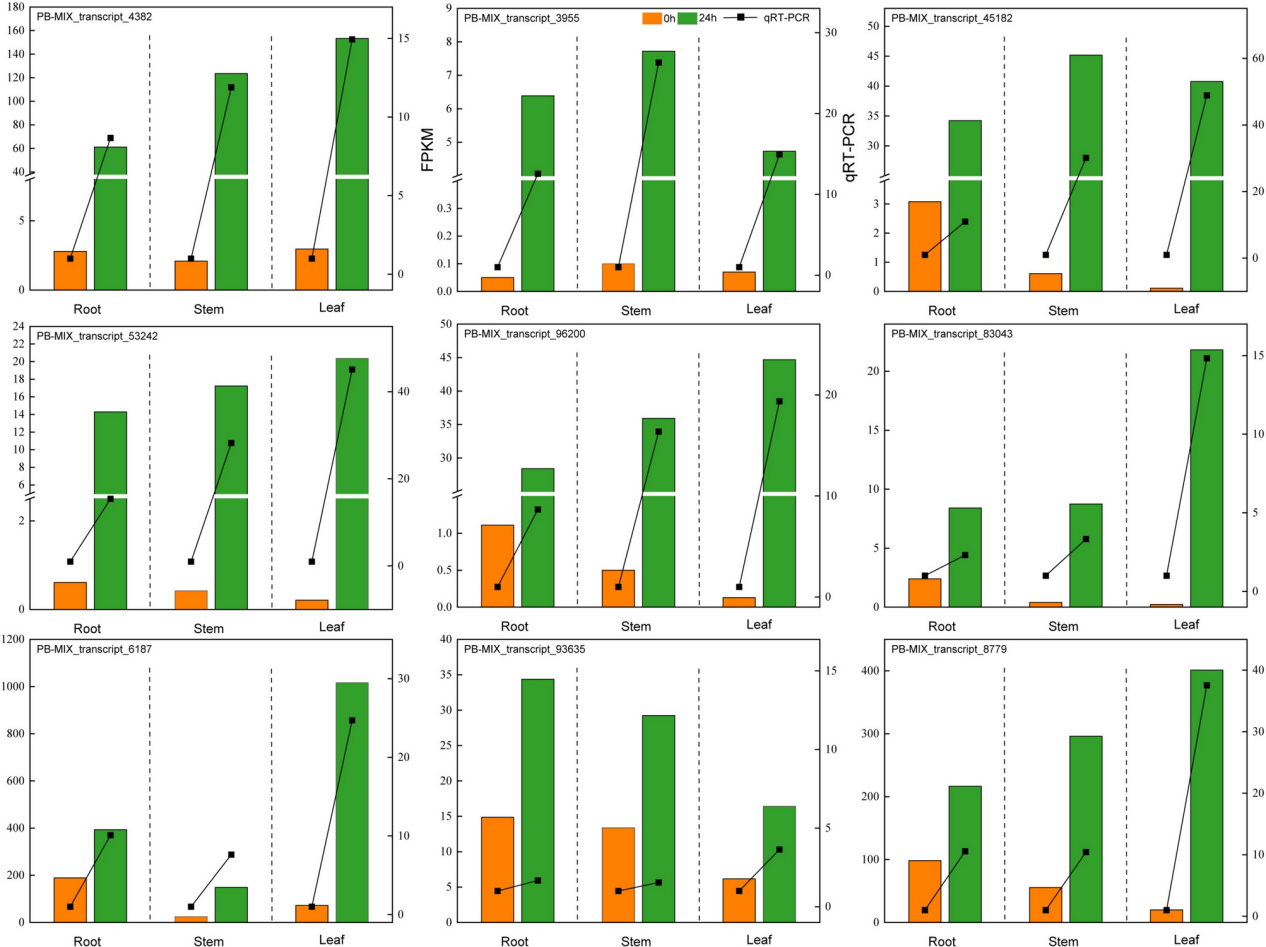
The qRT-PCR validation of RNA-Seq data. Note: The left vertical axis represents RNA-seq-FPKM, the right vertical axis represents qRT-PCR, and the horizontal axis represents different samples.

A total of 9 DETs were carefully chosen from key metabolic and signaling pathways, including “circadian rhythm in plants”, “arginine and proline metabolism”, “plant hormone signal transduction” as well as “starch and sucrose metabolism”. These DETs were selected for qRT-PCR analysis to validate the accuracy of the RNA-Seq data. As depicted in Fig. 7, the qRT-PCR results for the majority of these DETs across various organs of *A. bracteata* aligned well with the FPKM values from the RNA-Seq analysis. This concordance suggested that the RNA-Seq data obtained in this study was both accurate and reproducible.

## Discussion

The development and application of drought resistant tree species is a main way to enrich biodiversity in desert areas. *A. bracteata* as a typical shrub in desert areas, studying its drought resistance mechanism can provide scientific basis for its further development and application. In present study, we analyzed the full-length transcriptome and DETs in its roots, stems and leaves of *A. bracteata* under drought stress based on third-generation sequencing and combined with second-generation sequencing technology, morphological and physiological indicators. A total of 291 314 CCSs with mean read length of 2 062, and 248 178 FLNC, and 63 907 non-redundant FLNC were obtained (Table 1), meanwhile non-redundant FLNC sequences had an annotation rate of 86.96%. In addition, there were 2 821, 3 907 and 5 532 DETs identified from CKR vs. HR24h, CKS vs. HS24h and CKL vs. HL24h, respectively. In addition, as the degree of drought stress increased, the phenotype, osmotic substances, and antioxidant enzyme activity of different organs of *A. bracteata* undergo significant changes. It can be inferred that under drought stress, *A. bracteata* may alter the accumulation of related stress resistant substances and phenotypic changes by activating the expression of some drought resistant genes, and ultimately regulate its drought resistance.

RNA-Seq had become the most mature method for analyzing gene expression differences, RNA variable splicing phenomena, and miRNA regulatory effects in different periods and organs. However, RNA-Seq also had its own inevitable drawbacks, and which was difficult to accurately splice full-length transcripts and had more difficult to recognize long-chain non coding RNAs, fusion transcripts and homologous gene transcripts^50^. The Iso-Seq could directly reverse transcribe to obtain full-length cDNA, and which included a single complete transcript sequence information based on its ultra long reading, and subsequent analysis did not require assembly^31^. Because molecular biology research on *A. bracteata* has just started and there was no report on its genome, SMRT sequencing could provide a foundation for in-depth research in various aspects. In present study, using SMRT sequencing of *A. bracteata*, an entire of 31.86 Gb of clean data were acquired, and the numbers of CCS、FLNCs and non-redundant FLNC were also quite considerable compared to insects^41,51^, crops^36,52^ and trees^53–55^. That is to say, this study’s comprehensive transcriptome data might offer great support for the early molecular research of *A. bracteata.*

AS can play an important role on mechanism for gene expression regulation and can enhance the diversity of proteomes while having a smaller genome, adapting to the complexity of gene regulation requirements in higher multicellular organisms, it was also crucial in reacting to abiotic stress^56,57^. For example, the *Oryza Sativa* could improve drought resistance based on directly generate OsDREB2B2 by AS and spliced I1, E2, and I2 under drought stress^58^. Sanchez Martin et al. researched on the disease resistance of *Triticum aestivum,* and found that there were two ASs enhance its disease resistance by forming endoplasmic reticulum related complex^59^. In this study, we identified 2 093 AS events, but unfortunately, it could not classify them because of lacking of genomic data on *A. bracteata.*

TFs were a type of DNA binding proteins whose DNA binding domain (DBD) could specifically bind to specific sequences in the gene promoter region, thereby inhibiting or activating gene expression^60^. Furthermore, TFs were frequently involved in plant drought resistance, which focused on regulating the expression of genes related to drought response, mostly through activation or inhibition to regulate plant drought resistance. Among them, important transcription factors related to drought resistance include MYC/MYB, DREB, WRKY, bZIP, NAC and so on^61^. In present study, a total of 4 802 TFs were obtained from 64 families and the top 10 TF families were RLK-Pelle-DLSV, bHLH, others, MYB-related, C3H, AP2/ERF-ERF, bZIP, SNF2, WRKY and NAC, respectively, and most of them were related to plant stress resistance. NAC was unique to plants and played a key role in abiotic stress^62,63^. WRKY was a type of zinc finger transcription factor that participated in various abiotic stress responses^64,65^. As a regulatory protein, MYB was crucial for plants, and it widely participated in the response of plants to hormones and environmental factors^66^. In current study, when analyzed the function of DETs in different parts of *A. bracteata* under drought stress, the results showed that the “circadian rhythms-plant” pathway were significantly enriched in roots, stems and leaves. So, we further analyzed the circadian rhythm pathway which revealed that most of DETs mainly belonged to the MYB-related family. Therefore, we speculated that the TF of MYB-related played an important regulatory role in *A. bracteata* under drought stress. That is to say, TFs are quite necessary in studying the drought resistance of *A. bracteata,* and the TFs identified in present study could help *A. bracteata* to deal with drought stress through their complex regulatory.

The circadian rhythm is a biological rhythm that undergoes 24-h periodic changes when organisms are affected by various external environments^67,68^. When plants subjected to abiotic stress, their circadian rhythm also would have changed so as to enable them better to cope with environmental changes. The biological clock could transfer the valuable energy from responding to various stresses to plant growth by controlling various metabolic and physiological activities, which was the core of accurately synchronizing the circadian rhythm with the external environment to improve plant tolerance to abiotic stress^67^. When plants were subjected to drought stress, most drought response genes exhibited rhythmic expression patterns^69^, therefore drought was also regulated by circadian rhythms. For example, *PRR7* may be closely related to drought, and most ABA and drought related genes may be negatively regulated by *PRR7*, while plants overexpressing *PRR7* exhibited significant high sensitivity to drought stress^68,70,71^. In this study, DETs were significantly enrichment in the circadian rhythm-plant pathway, and there were 83 DETs, 86 DETs and 100 DETs in the CKR vs. HR24h, CKS vs. HS24h and CKL vs. HL24h, respectively. The majority of DETs participated in the circadian rhythm-plant pathway, including *PRR7*, were down-regulated in the roots, stems and leaves of *A. bracteata* following drought treatment, which were consistent with previous research results. In addition, scholars also found that *PRR7* was mainly participated in the response of oxidative stress and the regulation of stomata conductance^68,72^, which would better help us to research the drought resistance ability of *A. bracteata*.

To further analyze the mechanism of changes in physiological indicators of *A. bracteata* under drought stress, we analyzed the metabolic pathways of arginine and proline, and found that under severe drought stress, the number of DETs related to proline synthesis regulation genes (“*PRODH*, *fadM, putB*, *rocD*, *OAT*, *ALDH18A1*, *P5CS*, *P4HA*”) in different organs of *A. bracteata* changed, with the highest number in leaves and fewer in roots and stems, all of which were up-regulated. This is consistent with the accumulation trend of proline content measured by physiological indicators (Fig. 2B), indicating that under drought stress, the expression regulation of drought resistant genes and physiological responses in different organs synergistically regulate the drought tolerance strategy of *A. bracteata*. In addition, *PRODH* is a key enzyme gene in the degradation process of proline, and *P5CS* is a key enzyme gene in the synthesis of proline. A large number of studies have shown that under stress conditions, the expression of *P5CS* gene increased, while the expression of degradation enzyme gene *PRODH* decreased, leading to an increase in the synthesis of proline in plants and a significant accumulation, thus improving stress resistance^73^. In this study, both *P5CS* and *PRODH* genes were all up-regulated, which showed *A. bracteata* has its own drought resistance regulatory characteristics.

There were many metabolic pathways related to plant stress, such as “phenylpropane biosynthesis”, “flavonoid biosynthesis”, “photosynthesis”, “plant hormone signal transduction”, ect. Our research group had previously studied the “phenylpropane biosynthesis”, “plant hormone signal transduction” and “flavonoid biosynthesis” pathway which were significantly enriched in the leaves of different ages of *A. bracteata* under natural drought^74^. In present study, the metabolic pathways of “phenylpropane biosynthesis” and “flavonoid biosynthesis” were also significantly enriched in roots of *A. bracteata* under drought stress, but relatively less in stems and leaves. The “plant hormone signal transduction” pathway was significantly enriched in leaves of *A. bracteata*, but relatively less in stems and roots. Therefore, this study focused on analyzing the pathways significantly enriched in all organs of *A. bracteata*, and has not yet analyzed those pathways specifically enriched in various organs. In the future, it is necessary to further study the specific pathways significantly enriched in different organs of *A. bracteata* under drought stress, which will provide a basis for further exploration of functional genes.

## Conclusion

Under severe drought stress, the phenotype and physiological indicators of *A. bracteata* have undergone significant changes, and its DETs also have significant differences in different organs. In addition, the full-length transcriptome of *A. bracteata* under drought was sequenced and analyzed, which obtained a large of transcript data. Through analyzed the transcript raw data, main function and structure, we found that *A. bracteata* have large research space in terms of homologous species and new gene mining. Meanwhile, using the full-length transcriptome of *A. bracteata* as reference genome was help for studying the function of DETs under drought stress from RNA-seq. The selected DETs from different organs of *A. bracteata* were significantly enriched in the “starch and sucrose metabolism” and “circadian rhythm-plant” pathway, which maybe the key pathways in the gene regulation of *A. bracteata* under drought. Therefore, we could further explore some key genes for drought resistance of *A. bracteata* through these two pathways in the future research. In summary, this study could provide a reliable molecular basis and new ideas for future research on drought tolerance of *A. bracteata*.

## Supplementary Information

Supplementary Information 1.

Supplementary Figure1.

Supplementary Figure2.

Supplementary Legends.

Supplementary TableS1.

Supplementary TableS2.

Supplementary TableS3.

Supplementary TableS4.

Supplementary TableS5.

Supplementary TableS6.

Supplementary TableS7.

## Data Availability

The data of this study has been submitted to NCBI (https://www.ncbi.nlm.nih.gov/) and are publicly available at the number PRJNA 1 030 753 and PRJNA 1 028 835.
