# Supplementary figures and images for: Analysis of full length transcriptome and resistance characteristics of Atraphaxis bracteata under drought
Source: Sci Rep. 2025 Jan 4;15:807. doi: 10.1038/s41598-024-80831-2 (PMC11700114; doi:10.1038/s41598-024-80831-2)

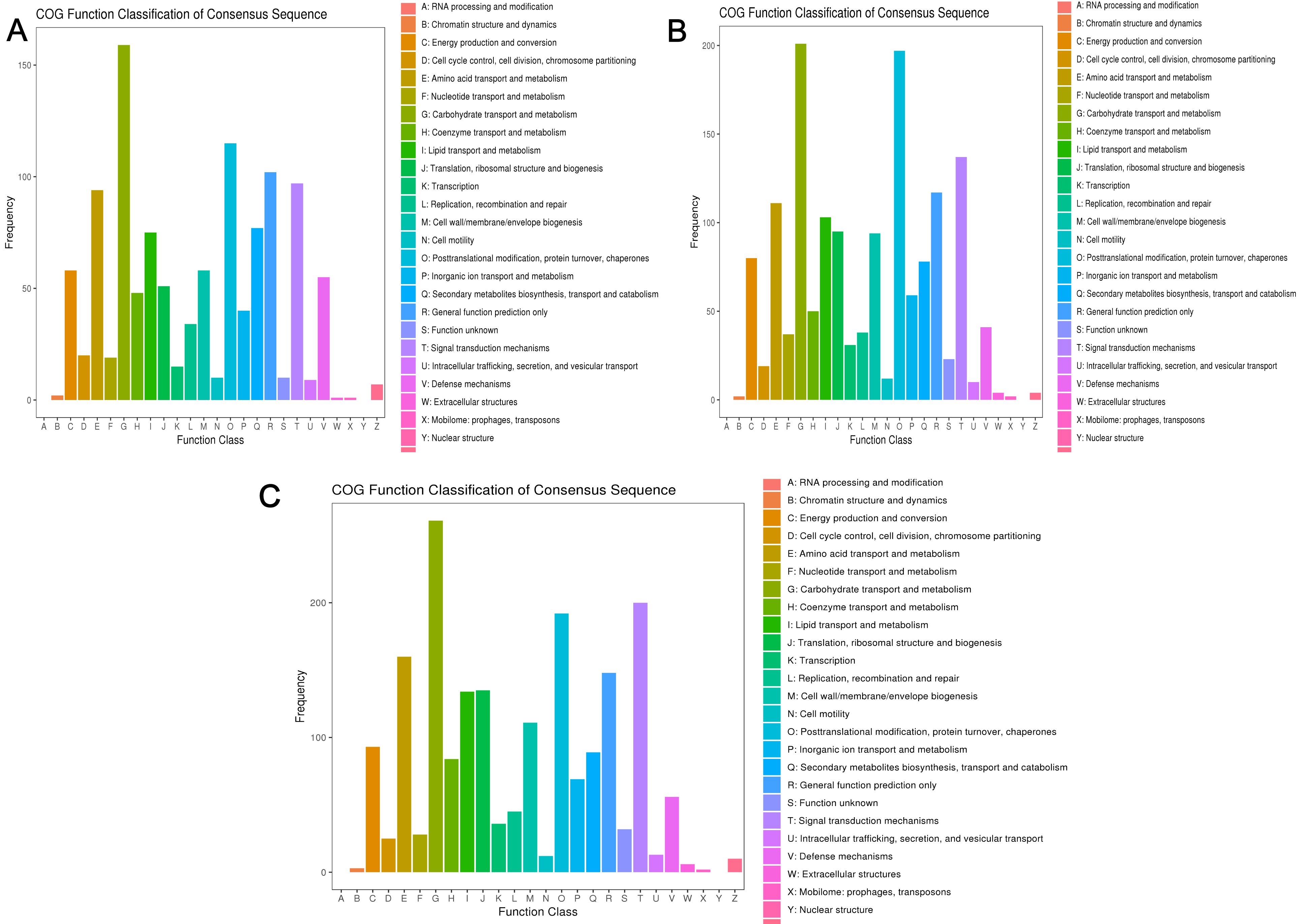

Supplement: Supplementary file 1 — Supplementary Information 1. [file 41598_2024_80831_MOESM1_ESM.zip › supplementary files/Supplementary Figure/Fig. S1.tif]

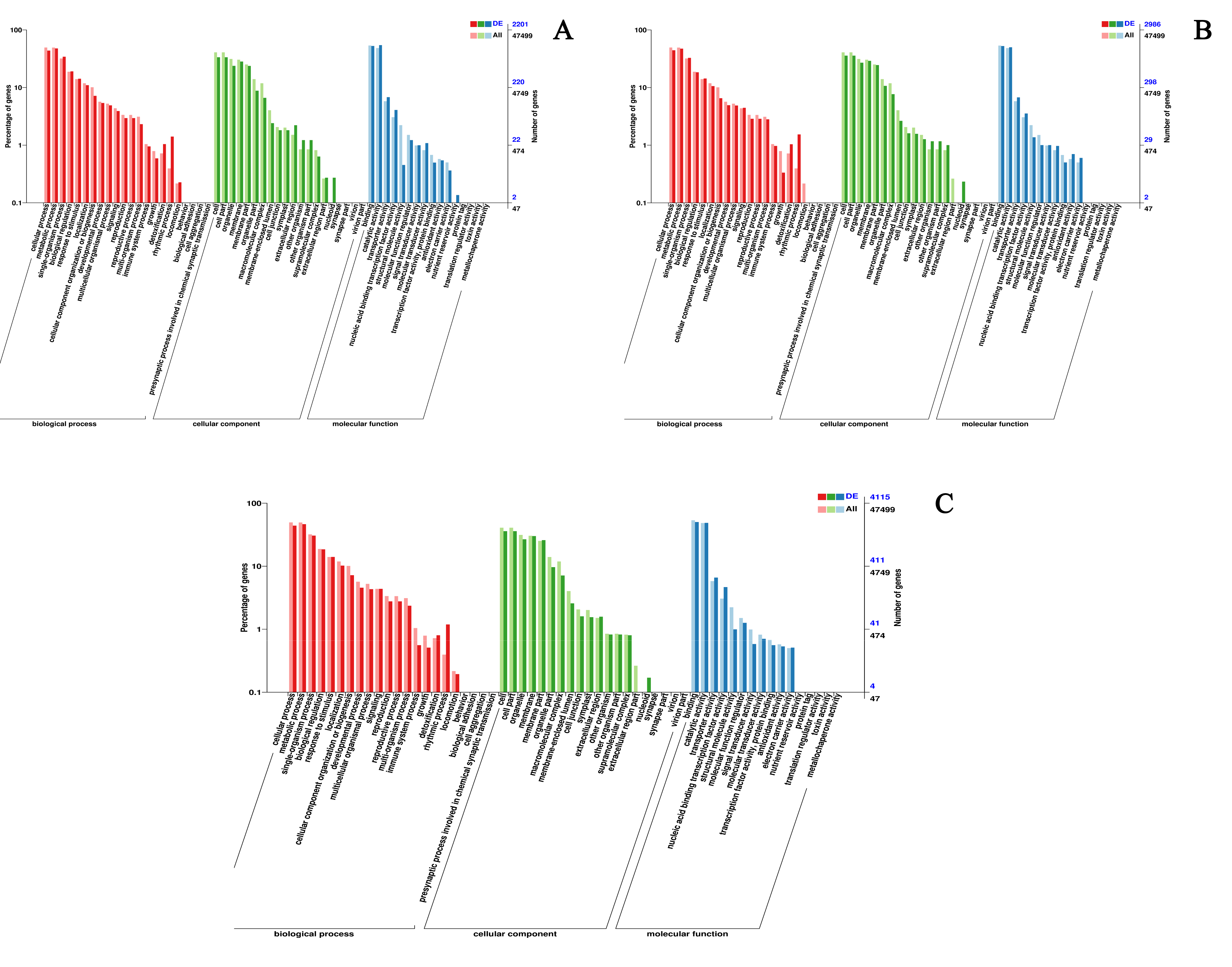

Supplement: Supplementary file 1 — Supplementary Information 1. [file 41598_2024_80831_MOESM1_ESM.zip › supplementary files/Supplementary Figure/Fig. S2.tif]
