# Supplementary material for: Analysis of full length transcriptome and resistance characteristics of Atraphaxis bracteata under drought
Source: Sci Rep. 2025 Jan 4;15:807. doi: 10.1038/s41598-024-80831-2 (PMC11700114; doi:10.1038/s41598-024-80831-2)
Supplement: Supplementary file 4 — Supplementary Legends. [file 41598_2024_80831_MOESM4_ESM.doc]

**Fig. S1.** The statistical diagram of COG annotation classification for differentially expressed transcripts (DETs). (A) The COG annotation classification statistics of DETs for CKR vs. HR24h. (B) The COG annotation classification statistics of DETs for CKS vs. HS24h. (C) The COG annotation classification statistics of DETs for CKL vs. HL24h.

**Fig. S2.** The statistical diagram of GO annotation classification for differential expressed transcripts (DETs). (A) The GO annotation classification statistics of DETs for CKR and HR24h. (B) The GO annotation classification statistics of DETs for CKS vs. HS24h. (C) The GO annotation classification statistics of DETs for CKL vs. HL24h.
